# Globally Rare *BRCA2* Variants With Founder Haplotypes in the South African Population: Implications for Point-of-Care Testing Based on a Single-Institution *BRCA1/2* Next-Generation Sequencing Study

**DOI:** 10.3389/fonc.2020.619469

**Published:** 2021-02-12

**Authors:** Jaco Oosthuizen, Maritha J. Kotze, Nicole Van Der Merwe, Ettienne J. Myburgh, Phillip Bester, Nerina C. van der Merwe

**Affiliations:** ^1^Division of Human Genetics, Faculty of Health Sciences, University of the Free State, Bloemfontein, South Africa; ^2^Division of Human Genetics, National Health Laboratory Service, Universitas Hospital, Bloemfontein, South Africa; ^3^Department of Pathology, Division of Chemical Pathology, Faculty of Medicine and Health Sciences, Stellenbosch University, Tygerberg, South Africa; ^4^Division of Chemical Pathology, National Health Laboratory Service, Tygerberg Hospital, Cape Town, South Africa; ^5^Panorama Centre for Surgical Oncology, Cape Town, South Africa; ^6^Division of Virology, National Health Laboratory Service, Universitas Hospital, Bloemfontein, South Africa

**Keywords:** *BRCA2*, founder variants, South Africa, breast cancer, next-generation sequencing, point-of-care assay

## Abstract

Breast cancer patients historically benefitted from population-based genetic research performed in South Africa, which led to the development of founder-based *BRCA1/2* diagnostic tests. With the advent of next-generation sequencing (NGS) technologies, the clinical utility of limited, targeted genetic assays were questioned. The study focused on mining NGS data obtained from an extensive single-institution NGS series (n=763). The aims were to determine (i) the prevalence of the most common recurrent/founder variants in patients referred for NGS directly; and (ii) to explore the data for inferred haplotypes associated with previous and potential new recurrent/founder variants. The identification of additional founder variants was essential for promoting and potentially advancing to rapid founder-based *BRCA1/2* point-of-care (POC) technology as a time- and cost-effective alternative. NGS revealed actionable *BRCA1/2* variants in 11.1% of patients tested (*BRCA1* – 4.7%; *BRCA2* – 6.4%), of which 22.4% represented variants currently screened for using first-tier targeted genetic testing. A retrospective investigation into the overall mutation-positive rate for an extended cohort (n=1906), which included first-tier test results, revealed that targeted genetic testing identified 74% of all pathogenic variants. This percentage justified the use of targeted genetic testing as a first-tier assay. Inferred haplotype analysis confirmed the founder status of *BRCA2* c.5771_5774del (rs80359535) and c.7934del (rs80359688) and revealed an additional African founder variant (*BRCA2* c.582G>A – rs80358810). A risk-benefit analysis using a questionnaire-based survey was performed in parallel to determine genetic professionals’ views regarding POC testing. This was done to bridge the clinical implementation gap between haplotype analysis and POC testing as a first-tier screen during risk stratification of breast and ovarian cancer patients. The results reflected high acceptance (94%) of *BRCA1/2* POC testing when accompanied by genetic counselling. Establishing the founder status for several recurrent *BRCA2* variants across ethnic groups supports unselected use of the BRCA POC assay in all SA breast/ovarian cancer patients by recent local and international public health recommendations. Incorporating POC genotyping into the planned NGS screening algorithm of the Department of Health will ensure optimal use of the country’s recourses to adhere to the set standards for optimal care and management for all breast cancer patients.

## Introduction

The development of hereditary breast cancer (BC) results in most cases, from highly penetrant pathogenic variants in several genes, of which the most frequently studied are *BRCA1* and *BRCA2*. Pathogenic variants present in these genes predispose to hereditary breast and ovarian cancer (HBOC) syndrome, with related cancers often described as being more aggressive compared to sporadic cancers. *BRCA1*-related tumors are more frequently negative for hormone receptors and of high grade, with *BRCA2*-related disease on average being of a higher histological grade than sporadic cases (1–5). *BRCA1/2* pathogenic variants predispose women to breast and ovarian cancer (OVC) (6, 7). The cumulative risk for *BRCA1/2* mutation carriers of developing BC to the age of 80 years has been approximated at 72% (95% CI 65–79%) and 69% (95% CI 61–77%), respectively. The risk for developing OVC is lower, at around 44% (95% CI 36–53%) for *BRCA1* and 17% (95% CI 11–25%) for *BRCA2* heterozygotes (8). Current management strategies for pathogenic mutation carriers range from intensified surveillance from a younger age to risk reduction surgery of the breasts and/or ovaries and include risk-reducing medications (9). Detection of inherited pathogenic variants in asymptomatic carriers allows for the development of appropriate management strategies to reduce cancer incidence and enable early detection, thus reducing mortality and improving quality of life.

The interesting history of sub-Saharan Africa has highlighted the populations of South Africa (SA) concerning the field of medical and population genetics. Due to various migration events, including European colonialism from predominantly north-western Europe, the indigenous expansion to the south, and admixture introduced mainly by slaves and laborers from southern Asia, various unique genetic signatures have been imprinted on its peoples. With genetic drift and natural selection, these major events have created uniquely admixed populations residing at Africa’s southern-most region. Their composition and heritage have incited various population studies that attempted to identify each group’s genetic architecture (10–14).

Over the past two decades, HBOC families in SA have derived great benefit from similar studies, which resulted in the development of a diagnostic, cost-effective first-tier genotyping assay based on a limited number of population-specific pathogenic *BRCA1/2* founder or recurrent variants. With the advent of low-cost next-generation sequencing (NGS) technologies, this assay’s clinical utility was questioned based on the SA populations’ collective genetic diversity. It caused a divergence from founder/recurrent variant testing to comprehensive *BRCA1/2* screening, which resulted in increased strain on the financially challenged health sector. Concerns were also raised that medical professionals and patients may misinterpret the exclusion of population-specific pathogenic *BRCA1/*2 variants as a negative test result.

This study focused on exploring the potential of a new genetic counselling model that incorporates rapid point-of-care (POC) *BRCA1/2* founder-based genotyping as a cost-effective alternative to SA’s current practices. Such a POC assay will allow for rapid clinical decision-making in mutation-positive patients and indicate extended NGS testing for deserving uninformative cases. Furthermore, this investigation relied on knowledge obtained regarding the incidence of founder variants in patients diagnosed with BC or OVC and the distribution of population-specific variants, including those not previously described. Thus, haplotypes associated with founder and recurrent *BRCA2* variants identified in the most extensive national, single-institution NGS series performed to date, were reconstructed. The confirmed founder/recurrent SA *BRCA2* pathogenic variants are suitable for inclusion in a customized DNA test kit developed under the South Africa-United Kingdom Newton Collaborative Research Development Program in Precision Medicine (https://gtr.ukri.org/projects?ref=103993). A current version of this kit was recently evaluated in a pilot study performed by Mampunye (15), highlighting the novel BRCA POC 1.0 Research Assay’s cost-saving potential. A qualitative survey was used as a first step towards assessing the thresholds that need to be overcome to bridge the clinical implementation gap between newly obtained research results and their incorporation into a POC assay.

## Materials and Methods

Samples of a total of 763 BC and/or OVC patients who attended various genetic clinics between 2017 and 2020 were received at the National Health Laboratory Service (NHLS) Human Genetics laboratory in Bloemfontein for comprehensive screening of *BRCA1/2* using NGS. Genomic DNA was isolated from peripheral blood (5–10 ml) using the salting-out method (16). The initial DNA quality was assessed with the NanoDrop® ND-1000 Spectrophotometer (NanoDrop® Technologies Inc., Wilmington, DE, USA), whereas the Qubit dsDNA High Sensitivity Assay kit was used to quantify DNA with the Qubit^®^ Fluorometer (Invitrogen; Thermo Fisher Scientific, Inc., Waltham, MA, USA) for NGS. Reference sequences used for *BRCA1* and *BRCA2* analyses were GenBank NM_007294.3 (*BRCA1*) and NM_000059.3 (*BRCA2*).

NGS was performed using the Oncomine™ BRCA Research Assay (Life Technologies, Carlsbad, CA, USA). The primer pools targeted the entire coding region together with intronic flanking sequences for both genes. The amplicon library was constructed using multiplexed primer pools during PCR-based targeted amplification. Sequencing was performed on the Ion Proton Platform (Life Technologies, Carlsbad, CA, USA) and the Ion Reporter™ Software (Life Technologies, Carlsbad, CA, USA) used to filter out possible artifacts. Raw signal data were analyzed using the Torrent Suite™ versions 5.2 to 5.14.

Genotyping for the most common SA pathogenic variants (17) was performed as a first-tier test (n=1906) for all breast and OVC patients. It was performed on the LightCycler^®^ 480 II instrument (Roche Diagnostics Applied Science, Mannheim, Germany) using hybridization probe technology for six of the variants (*BRCA1* c.68_69delAG, p.Glu23ValfsX17; *BRCA1* c.1374delC, p.Asp458Glufs; *BRCA1* c.2641G*>*T, p.Glu881Ter; *BRCA1* c.5266dupC, p.Gln1756Profs; *BRCA2* c.7934delG, p.Arg2645Asnfs) and two simple probe assays for *BRCA2* c.5771_5774del, p.Ile1924Argfs and *BRCA2* c.6448_6449dup, p.Lys2150fs. The primer and probe sequences have been listed by Oosthuizen (18). Each qPCR reaction contained 50 ng genomic DNA, 3 µM of each primer (TIB MolBiol, Berlin, Germany), 2 µM of each probe (TIB MolBiol), 4 µl LightCycler^®^ 5X Genotyping Master Mix (Roche Diagnostics GmbH, Mannheim, Germany), together with 12.6 µl molecular grade H_2_O. A standard qPCR regime was utilized, followed by a melt curve acquiring fluorescence data at a frequency of 5 readings per °C to determine the melting point (T_m_) (18). Each variant was tested for individually, together with a positive, negative and no template control to ensure sensitivity and specificity. The genotyping reports generated over the years were retrospectively analyzed to evaluate the assay’s success as a first-tier test (**Table 1**) (19).

**Table 1:** Detection rates for the most common SA pathogenic variants included in the first-tier genotyping assay, according to ethnicity and clinical diagnosis.

First-tier pathogenic variants	Ethnicity^b^	Number of patients tested	Number of negative results	Number of positive results	Detection rate
NM_007294.3(*BRCA1*):c.1374delC p.Asp458Glufs	Afrikaner	758	749	9	1.2%
		Affected: 436	433	3	0.7%
		Pre-symptomatic: 322	316	6	1.9%
NM_007294.3(*BRCA1*):c.2641G>T p.Glu881Ter	Afrikaner Coloured^a^	758	733	25	3.3%
		Affected: 436	424	12	2.8%
		Pre-symptomatic: 322	309	13	4.0%
		600	597	3	0.5%
		Affected: 537	534	3	2.8%
		Pre-symptomatic: 63	63	0	0.0%
NM_000059.3(*BRCA2*):c.7934delG p.Arg2645Asnfs	Afrikaner Coloured^a^	758	623	135	17.8%
		Affected: 436	351	85	19.5%
		Pre-symptomatic: 322	272	50	15.5%
		600	597	3	0.5%
		Affected: 537	584	16	2.7%
		Pre-symptomatic: 63	61	2	3.2%
NM_000059.3(*BRCA2*):c.5771_5774del p.Ile1924Argfs	Coloured^a^ Black	600	587	13	2.2%
		Affected: 537	527	10	1.9%
		Pre-symptomatic: 63	60	3	4.8%
		548	508	40	7.3%
		Affected: 521	487	34	6.5%
		Pre-symptomatic: 27	21	6	22.2%
**Total**		**1906**	**1665**	**241**	**12.6%**

^a^Mixed ethnicity; ^b^Only the ethnic groups in which the respective variants were detected are listed, as the variants are population-specific.

All patients included in the NGS cohort (n = 763) were born in the country and represented the SA population. They were selected for comprehensive screening by their healthcare professionals based on their diagnosis with either breast or OVC at an early age (<40 years) or the presence of a personal and family history of the disease. All the patients underwent pre- and post-test counselling at the respective referring national hospitals. Information regarding a personal and/or family history of the disease, together with written informed consent for testing, was provided. The cohort included medium- (two affected family members) to high-risk families (>3 affected family members), with most representing low-risk patients who had no family history of either condition but was diagnosed at an early age of onset (<40 years). Population group was determined by patient self-identification and represented all main SA ethnic groups. The Ethics Committee of the Faculty of Health Sciences at the University of the Free State, together with the Health Research Ethics Committee of Stellenbosch University, approved all study procedures (UFS-HSD2019/1835/2910, UFS-HSD2020/0194/3006, US-N09/08/224) and the NHLS permitted use of the data.

Inferred haplotype analysis was performed for the four most prevalent *BRCA2* pathogenic variants to determine the presence of possible founder effects (**Table 2**). Haplotypes that were positively associated with these internationally rare *BRCA2* pathogenic variants would support a potential founder effect. Genotypes based on multiple SNPs retrieved from the NGS data were compared among patients carrying a specific pathogenic variant (n≥5) and checked against a reference haplotype constructed by using mutation-negative individuals.

**Table 2:** Details of the most common *BRCA2* pathogenic variants observed and their recurrence internationally.

*BRCA2* variant		rs ID	HGVS^a^	ClinVar	PAGE study	Current SA study^b^
DNA level	Protein level					
c.582G>A	p.Trp194Ter	rs80358810	NC_000013.10:g.32900701G>A	5	3	11
c.5771_5774del	p.Ile1924fs	rs80359535	NC_000013.10:g.32914263_32914266del	5	0	45
c.6447_6448dup	p.Lys2150fs	rs397507858	NC_000013.10:g.32914939_32914940dup	6	0	7
c.7934del	p.Arg2645Asnfs	rs80359688	NC_000013.10:g.32936788delG	10	0	161

^a^The variants are defined according to the Human Genome Variation Society guidelines; ^b^The numbers indicated include both pre-symptomatic carriers and affected individuals. Genomic positions are according to the GRCh37/h19 human genome build.

Linkage disequilibrium (LD) analysis was performed to construct reference haplotypes for *BRCA2*, using the NGS data. The process commenced with the identification of SNPs and their associated minor allele frequencies (MAF). This step was necessary to eliminate rare variants unique to individuals that could weaken the LD analysis and prevent the reconstruction of haplotype blocks assorting independently as determined by contingency χ2. A total of 36 SNPs were selected for LD analysis based on a MAF > 0.001 in the SA population. The SNP identification codes and genomic positions based on the GRCh37/h19 human genome build are listed in **Table 3**. The selected SNPs were distributed across 82 kb and were situated mainly in the exons and exon/intron boundaries.

**Table 3:** Complete list of *BRCA2* SNPs detected by means of NGS amongst the mutation carrier and control cohorts.

SNP number	rs ID	Variant name	Chromosome Position	Global MAF ALFA Global: 183,188 chromosomes	MAF in SA population	MAF of variants included in haplotype (MAF>0.01) African: 6656 chromosomes European: 159208 chromosomes South Asian: 4904 chromosomes
SNP1	rs1799943	c.-26G>A	chr13:32890572	0.256	0.121	African: 0.110
						European: 0.265
						South Asian: 0.291
SNP2	rs76874770	c.-11C>TA	chr13:32890587	0.004	0.02	African: 0.017
						European: 0.000
						South Asian: 0.000
SNP3	rs81002794	c.317-22C>T	chr13:32899191	0	0.009	Excluded
SNP4	rs81002804	c.517-4C>G	chr13:32900632	0	0.028	African: 0.000
						European: 0.000
						South Asian: 0.000
SNP5	rs80358810^a^	c.582G>A	chr13:32900701	0	0.005	Excluded
SNP6	rs2126042	c.681+56C>T	chr13:32903685	0.186	0.226	African: 0.243
						European: 0.185
						South Asian: 0.124
SNP7	rs144848	10: c.1114A>C	chr13:32906729	0.279	0.216	African: 0.149
						European: 0.283
						South Asian: 0.339
SNP8	rs750755676	11: c.2299A>C	chr13:32910791	0	0.001	Excluded
SNP9	rs1801406	11: c.3396A>G	chr13:32911888	0.311	0.178	African: 0.248
						European: 0.314
						South Asian: 0.305
SNP10	rs543304	11: c.3807T>C	chr13:32912299	0.182	0.169	African: 0.188
						European: 0.184
						South Asian: 0.114
SNP11	rs80359406	11: c.3858_3860delAAA	chr13:32912345	0	0.004	Excluded
SNP12	rs41293485	11: c.3869G>A	chr13:32912361	0	0.01	African: 0.013
						European: 0.000
						South Asian: 0.000
SNP13	rs56248502	11: c.4090A>C	chr13:32912582	0	0.024	African: 0.015
						European: 0.000
						South Asian: 0.000
SNP14	rs545444016	11: c.4502A>G	chr13:32912994	0	0.005	Excluded
SNP15	rs206075	11: c.4563A>G	chr13:32913055	0.988	1	African: 0.929
						European: 0.998
						South Asian: 1.000
SNP16	rs55639415	11: c.5198C>T	chr13:32913690	0	0.009	African: 0.001
						European: 0.000
						South Asian: 0.000
SNP17	rs80358765	11: c.5414A>G	chr13:32913906	0	0.027	African: 0.000
						European: 0.000
						South Asian: 0.000
SNP18	rs80359535^a^	11: c.5771_5774del	chr13:32914260	0	0.003	Excluded
SNP19	rs11571659	11: c.6412G>T	chr13:32914904	0	0.009	African: 0.002
						European: 0.000
						South Asian: 0.000
SNP20	rs397507858^a^	11: c.6447_6448dup	chr13:32914939	0	0.003	Excluded
SNP21	rs206076	11: c.6513G>C	chr13:32915005	0.996	0.998	African: 0.956
						European: 0.999
						South Asian: 1.000
SNP22	rs1799955	14: c.7242A>G	chr13:32929232	0.213	0.149	African: 0.229
						European: 0.219
						South Asian: 0.170
SNP23	rs169547	14: c.7397T>C	chr13:32929387	0.997	0.978	African: 0.941
						European: 0.999
						South Asian: 1.000
SNP24	rs56070345	15: c.7505G>A	chr13:32930634	0	0.001	Excluded
SNP25	rs9534262	17: c.7806-14T>C	chr13:32936646	0.514	0.484	African: 0.554
						European: 0.515
						South Asian: 0.484
SNP26	rs80359688^a^	17: c.7934delG	chr13:32936787	0	0.009	Excluded
SNP27	rs81002827	17: c.7976+12G>A	chr13:32936842	0	0.005	Excluded
SNP28	rs146430937	18: c.8010G>A	chr13:32937349	0	0.005	Excluded
SNP29	rs80359052	18: c.8092G>A	chr13:32937431	0	0.009	African: 0.000
						European: 0.000
						South Asian: 0.000
SNP30	rs28897747	18: c.8149G>T	chr13:32937488	0.001	0.002	African: 0.000
						European: 0.001
						South Asian: 0.000
SNP31	rs81002808	19: c.8332-66T>C	chr13:32944473	0	0.009	African: 0.010
						European: 0.000
						South Asian: 0.000
SNP32	rs11571744	21: c.8487+47C>T	chr13:32944741	0	0.032	African: 0.010
						European: 0.000
						South Asian: 0.000
SNP33	rs4942486	22: c.8755-66T>C	chr13:32953388	0.512	0.429	African: 0.501
						European: 0.514
						South Asian: 0.484
SNP34	rs4987047	22: c.8830A>T	chr13:32953529	0.001	0.036	African: 0.030
						European: 0.000
						South Asian: 0.000
SNP35	rs56121817	27: c.9875C>T	chr13:32972525	0	0.008	African: 0.000
						European: 0.000
						South Asian: 0.000
SNP36	rs1801426	27: c.10234A>G	chr13:32972885	0.007	0.06	African: 0.088
						European: 0.001
						South Asian: 0.000

^a^Pathogenic variants not included in the haplotype illustrated in **Figure 1**; Global and continental MAF reference according to NCBI and ALFA Release Version: 20200227123210 (20).

**Figure 1:**
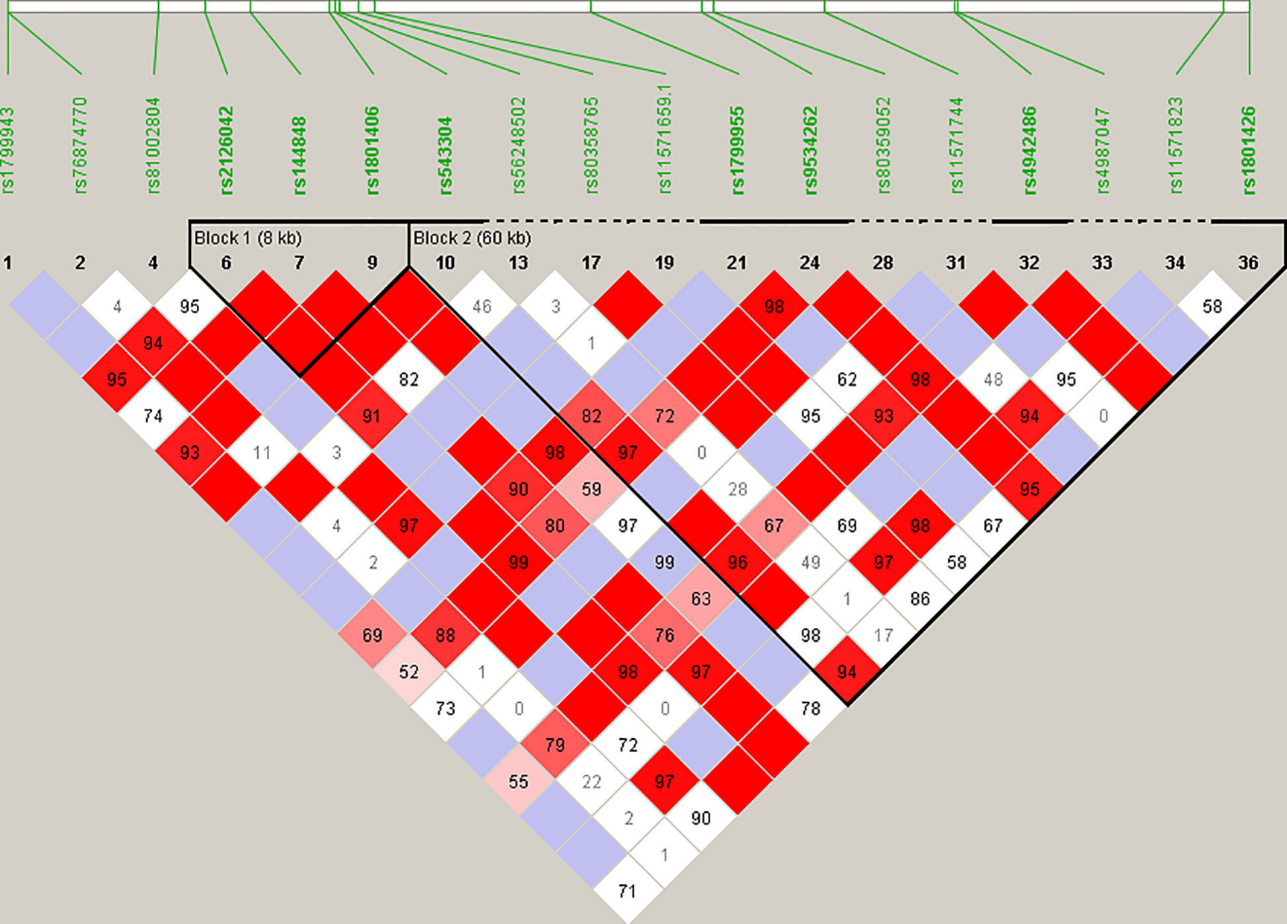
Linkage disequilibrium (LD) plot constructed for *BRCA2* with Haploview 4.2, using a total of 18 SNPs. Schematic diagram of the gene on chromosome 13. Marker variants and their relative locations are represented by vertical lines or boxes (SNP number is indicated above each box together with the rs ID). The LD plot is based on the logarithm of the odds (LOD score) and average obsolete value (D’) to characterize the LD between two SNPs in the population data. The diamond color where two SNPs intersect reflected the LD’s level, with bright red indicating very strong LD (LOD = 2, D’ = 1), a white color for no LD (LOD < 2, D’ < 1), with pink-red (LOD = 2, D’ < 1) and blue (LOD < 2, D’ = 1) for an intermediate LD.

Haplotype blocks were constructed using Haploview 4.2 (https://www.broadinstitute.org/haploview/haploview) (21). The program created an LD plot (**Figure 1**) using the logarithm of the odds (LOD score) and average obsolete value (D’) between two SNPs. Built-in quality checks of the software resulted in the exclusion of 18 SNPs based on a MAF < 0.01 and deviation from the Hardy Weinberg equilibrium. Haplotype blocks were constructed according to the algorithm and block definitions stipulated by Gabriel et al. (22). Using Haploview 4.2, the diamond color where two SNPs intersect reflected the LD’s level, with bright red indicating very strong LD (LOD = 2, D’ = 1), white color for no LD (LOD < 2, D’ < 1), with pink-red (LOD = 2, D’ < 1) and blue (LOD < 2, D’ = 1) for an intermediate LD. The *BRCA2* pathogenic variants and their observed genotypes were assigned to predicted haplotypes through association frequency after LD analysis and haplotype block construction.

Before incorporating newly obtained results into a POC BRCA assay, the expressed demand for it to be used as a first-tier assay was assessed as part of a risk-benefit analysis through a survey published on the Open Genome Project website (https://www.gknowmix.org/opengenome/survey/). The survey distributed among SA genetics healthcare professionals also explored the most appropriate clinical setting within which such an assay should be performed. Responses from these professionals who attended the South African Society for Human Genetics conference held in Cape Town in 2019 were evaluated after excluding six questions and answering data sets that were considered irrelevant to the current study. The remaining questions were divided into two groups, relating to perceived benefits and risks.

## Results

Of the 763 patients screened using NGS, 85 (11.1%) carried a likely- to pathogenic *BRCA1/2* SNV (*BRCA1* 36/763, 4.7% and *BRCA2* 49/763, 6.4%). The mutation rates differed among the ethnic groups, with 13 variants detected for the SA Indian (13/142, 9.1%; 7 in *BRCA1* and 6 in *BRCA2*), 13 Coloured individuals of mixed ancestry (13/120, 10.8%; 4 *BRCA1* and 9 *BRCA2*), 22 White Afrikaners (22/124, 17.7%; 11 *BRCA1* and 11 *BRCA2*), 35 Black patients (35/379, 9.2%; 13 *BRCA1* and 22 *BRCA2*) and two *BRCA1* variants in the non-Afrikaner White population (2/30, 6.7%). The rates detected for the Afrikaner and the Black populations included 11 and six patients, respectively, carrying previously described founder variants, generally excluded using the first-tier genotyping assay. The mutation rates for copy number variants were reported elsewhere (23). Of the 85 pathogenic variants detected, 19 (22.4%) represented variants included in the first-tier genotyping assay (*BRCA1* c.68_69delAG – rs80357783, 1.2%; *BRCA1* c.2641G>T – rs397508988, 1.2%; *BRCA2* c.5771_5774delTTCA – rs80359535, 7.1%; *BRCA2* c.7934delG – rs80359688, 12.9%).

**Figure 2:**
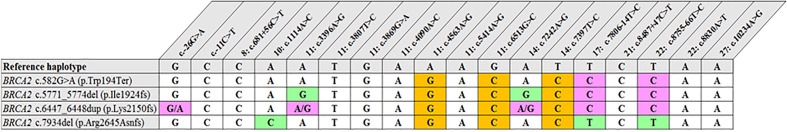
Shared haplotype of recurrent pathogenic variants. The core haplotype associated with each variant is represented by 18 SNPs spread throughout *BRCA2*. A core haplotype was observed for three of the four pathogenic mutation carriers representing a specific, actionable variant but was inconsistent in the control chromosomes. The pink blocks represent an alternative allele to that of the reference, whereas the blocks highlighted in green indicate unique genotypes associated with the specific variant.

**Figure 3:**
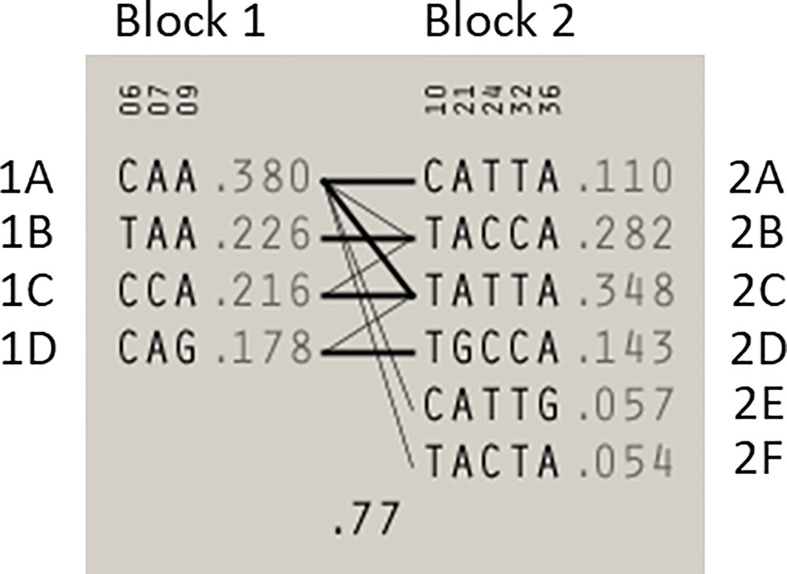
Haplotype blocks and their associated allele frequencies constructed for *BRCA2* with Haploview 4.2 using eight SNPs with a MAF > 0.01 with a strong LD. Schematic diagram of the two blocks. The multi-locus D’, which measures the LD between two blocks, is indicated below the crossing lines. Thick connection lines represent haplotype block recombinations observed >10%, whereas a thin connection line represents haplotype block recombination >1%.

Statistical reconstruction of reference haplotypes was performed using 18 SNPs, with MAF > 0.01. Haplotype analysis showed that the SNPs segregated in two LD blocks (>95% probability), encompassing eight SNPs in strong LD (**Figure 2**). The blocks consisted of an 8 kb segment (block 1: rs2126042–rs1801406) and a 60 kb segment (block 2: rs543304–rs1801426) (**Figure 2**). The blocks encompass eight SNPs in strong LD (LOD ≥ 2, D’ = 1), with three indicated in block 1 and five in block 2 (**Figure 2**). Block 1 consisted of four alleles, whereas block 2 indicated six alleles (multi-locus D’ = 0.77; **Figure 3**). Block 1A was involved in the most recombination events and was, therefore, the least conserved. This was in stark contrast to block 1B, which exhibited no recombination upon a well-conserved haplotype. Recombination between block 1B and 2B represented the most common haplotype (0.22). The lowest level of recombination was observed between block 1C and 2B (**Figure 3**). All the observed associations accounted for 96% of the haplotypes observed, indicating several unknown events present in the SA population, possibly involving rare SNPs (MAF < 0.01).

Most of the SNPs observed among mutation carriers representing the four pathogenic variants listed in **Table 2** were rare, with MAF < 0.01 (ALFA: Allele Frequency Aggregator) (20) (**Table 3**). Only eight SNPs had a MAF > 0.01 (**Table 3**). From the low frequencies indicated on ALFA, it is clear that most variants excluded proved to be unique to the African continent or SA individuals (**Table 3**). These differences in MAFs reflected the diversity of the SA population. Despite the low MAF scores for the majority of SNPs, a segregating haplotype was associated with three of the four pathogenic variants, namely *BRCA2* c.582G>A (based on seven affected mutation carriers compared to controls, haplotype 1A2B), *BRCA2* c.5771_5774delTTCA (n=8, haplotype 1D2D), and *BRCA2* c.7934delG (n=11, haplotype 1C2C) (**Figure 3**). These haplotypes were based on the allelic combinations observed at 18 markers (**Figure 3**). This confirmed the previous founder status classification of *BRCA2* c.7934delG and *BRCA2* c.5771_5774delTTCA based on genealogy (>10 generations) and phased microsatellite markers (24). For *BRCA2* c.6447_6448dup, the alleles observed at three distinct loci (*BRCA2* c.-26G>A, *BRCA2* c.3396A>G and *BRCA2* c.7242A>G; **Figure 3**) were not common amongst carriers of this variant. This finding resulted in the variant being classified as recurrent rather than a founder variant. Therefore, haplotype analysis confirmed a single additional founder variant in the Black SA population, namely *BRCA2* c.582G>A (rs80358810). This variant has not yet been included in the BRCA 1.0 POC Research Assay.

**Table 4** shows an extract from the qualitative survey results obtained from genetic professionals regarding the appropriateness for performing a first-tier genetic test in the form of the novel BRCA 1.0 POC Research Assay. This newly-developed assay currently includes all eight common SA variants screened for by the diagnostic laboratories of the National Health Laboratory Service and several private laboratories in SA (19), but has the potential to be more cost-effective and less time consuming when compared. The vast majority (94%) of survey participants indicated that it would be very convenient to have a rapid, affordable POC test available that can alter patient care with regard to clinical intervention and genetic counselling support. While 75% of participants argued that founder mutation analyses might be used widely in government hospitals as a first-tier test, only 9% reported that it would be used in private practice as gene panel testing is more often requested. However, 91% of stakeholders agreed that when patients (setting unspecified) cannot afford HBOC panel testing, targeted genetic testing will be better than no testing, keeping in mind the limitations of population-based testing. With regards to diagnostic and predictive *BRCA1/2* testing, 81 and 84% of participants, respectively, expressed concerns about the associated psychosocial impact of results made available within the hour or on the same day.

**Table 4:** Survey results indicating the responses of 32 workshop participants to statements relating to BC management including diagnostic and treatment-related *BRCA1/2* POC genetic testing.

B/R	Questions related to benefits (B) and risks (R)	Yes	N/A	No
B	It will be very convenient to have a rapid, affordable POC test available that can alter patient care with access to clinical intervention and genetic counselling support.	30 (94%)	0	2 (6%)
B	POC screening for founder mutations is important in the context of ancestry and family history.	25 (78%)	3	4 (13%)
R	The detection rate of *BRCA1/2* founder mutations is reducing due population diversification, therefore a population specific POC test will not be useful.	14 (44%)	4	14 (44%)
B	Often the report is provided when the patient has already started therapy or had surgery and that defeats the purpose of genetic testing.	11 (34%	0	21 (66%)
B	When patients cannot afford panel testing founder mutation testing will be better than nothing, knowing the limitations related to population-based testing.	29 (91%)	1	2 (6%)
R	From a private practice perspective, a *BRCA1/2* POC test will not be widely used as founder mutation testing is hardly ever requested anymore.	21 (66%)	8	3 (9%)
B	Founder mutation analyses are still first line testing for many conditions in the state sector and may therefore be used widely in government hospitals.	24 (75%)	0	8 (25%)
R	When a patient is going to pay for a genetic test out of pocket, most of the patients would prefer more comprehensive cancer gene panels the first time around rather than having to do more than one test later.	28 (88%)	2	2 (6%)
R	With regards to *BRCA1/2* predictive testing, the waiting period for results is helpful in giving the patient’s time to mentally prepare for the results.	27 (84%)	4	1 (3%)
R	Same day delivery of *BRCA1/2* results might be a bit daunting as these results have major implications with regards to the patients themselves, their reproductive choices and their children.	26 (81%)	0	6 (19%)
B	A missed genetic diagnosis of HBOC* is unlikely with the use of a combination of tests ranging from a rapid POC diagnostic assay for known *BRCA1/2* pathogenic mutations to MinION/whole genome sequencing using an integrated service and research approach for return of results.	25 (78%)	5	2 (6%)
R	Genetic counselling is essential for POC genetic testing that may require extension to clinical sequencing when the results are uninformative.	30 (94%)	1	(3%)

*HBOC, hereditary breast and ovarian cancer.

## Discussion

The results obtained from this single-institution NGS series delivered a positive mutation rate of 11.1%. Of the 85 mutation-positive patients, 17 patients (20%) carried one of the eight most common SA founder or recurrent pathogenic variants. Compared to the overall mutation-positive rate for the extended cohort (n=1906), targeted genetic testing identified 74% of all the pathogenic variants detected (241 of 326, including those detected by NGS, **Table 1**). These results indicate that performing targeted genetic testing as a first-tier assay remains extremely valuable for the country’s financially depleted healthcare system. By performing it for all affected breast and OVC patients, irrespective of cancer in the family or ethnic group, most familial variants will still be identified at a fraction of the costs involved with comprehensive screening. This observation corresponds to the findings of various international studies performed on founder populations such as French-Canadian (25) and Ashkenazi Jewish (26) groups. The data obtained from these studies justified a place for cost-effective targeted genetic testing for founder variants and even future population-based screening for cancer predisposition.

Our results mimic the recommendations of the NCCN in the United States, which state that standard care for all Ashkenazi Jewish individuals starts with screening for founder variants first (27). Using a similar approach will improve the results obtained with risk prediction tools such as the Manchester scoring system in the SA population. The inclusion of founder variant status will enhance the predictive score of a *BRCA1/2* variant being present. This is in line with our risk-benefit analysis based on 12 issues addressed in the needs-assessment survey, which provided useful information for paving the way forward (**Table 4**).

The two recurrent and two SA founder variants investigated are internationally rare (**Table 3**). *BRCA2* c.582G>A in exon 7 is located in an area of the gene (c.517 to c.587) that has global splicing enhancer properties (28). This area is known for harboring both the highest density of exonic splicing enhancers and the lowest density of exonic splicing silencers. This exon is therefore very sensitive to nucleotide variants affecting potential exonic splicing regulatory elements (29). The variant was initially reported by Francies et al. (30) in a single SA patient and later by Chen (31). This variant also represented one of the causative variants reported for a SA Black Fanconi anemia infant reported by Feben et al. (24). The variant was confirmed as a new SA founder variant based on the SNP haplotype analysis results.

The recurrent variant *BRCA2* c.6447_6448dup in exon 11 (historically known as *BRCA2* 6676insTA, rs397507858) entailed the duplication of two base pairs and was first described by Meindl in 2002 (32). The variant is globally rare and results in a null variant, directly affecting the associated protein. It was detected in eight self-identified Coloured patients (17, 18, 33). The age at onset/diagnosis in these patients varied from 27 to 63 years, with an average age of 49.2 years. Different genotypes were observed in mutation carriers at three loci, namely c.-26G>A, c.3396A>G and c.7242A>G (**Figure 1**), which resulted in its proposed classification as a recurrent variant. This finding is noteworthy given its current restriction to a single SA population group despite apparent uncertainty of the exact insertion/deletion position at a potential *BRCA2* mutational hotspot. This variant was initially listed by Agenbag (33) as *BRCA2* c.6449_6450insTA, and as c.6448_6449dupTA by Van der Merwe et al. (17) and Oosthuizen (18), despite corresponding electropherograms. Based on the new Human Genome Variation Society guidelines (http://www.HGVS.org/varnomen), this variant is currently officially known as *BRCA2* c.6447_6448dup. This entails noting the nucleotide number of the two base pairs involved in the duplication (nt 6447 and nt 6448) and not the location where the repeat was inserted.

*BRCA2* c.5771_5774del, historically known as *BRCA2* 5999del4, represents the most common pathogenic variant observed in both the Black and Coloured populations (**Table 1**). This variant is absent in the Afrikaner and SA Indian population and was identified seven times during the NGS study (7/763, 0.92%). The majority of the patients (n=5) were Black, with two patients who self-identified as Coloured. All the mutation carriers were diagnosed with BC, with most diagnosed ≤40 years (range 35–53 years). The deletion is predicted to cause loss of normal protein function through either protein truncation or nonsense-mediated mRNA decay. The variant occurred in the BRC domain (aa1009–2083) that facilitates the binding of RAD51 (34). This variant currently forms part of the first-tier genotyping assay that precedes comprehensive NGS analysis. The variant was detected collectively 53 times (53/1906, 2.8%), mostly in patients from the Western Cape (**Table 1**). The age at onset for mutation carriers ranged from 25 to 71 years, with many patients not reporting a family history of cancer. The seven BC patients included for the haplotype analysis represented both the Black and Coloured ethnic groups. Although a unique haplotype was observed, there was no distinction between patients representing each of these groups. The single-base deletion in exon 17 (*BRCA2* c.7934delG – rs80359688, historically known as *BRCA2* 8162delG) represents the most common Afrikaner founder variant, also included in the current first-tier genotyping assay (17, 35). Founder status was previously proven by genealogical and haplotype analysis using flanking and intragenic microsatellite markers (17, 35). The genealogical study involved 12 independent families linked to the variant, mapped over a minimum of 10 generations (data not shown). A total of 151 mutation carriers were identified, with 99 of them being affected with cancer (151/1906, 7.9%). It represents the country’s most common recurrent variant. It accounts for most BC and/or OVC patients in two populations, namely the Afrikaner and Coloured populations (**Table 1**) (19). The variant is located in the gene’s helical domain (oligonucleotide/oligosaccharide-binding fold OB1) responsible for the binding single- and double-stranded DNA (36, 37). The age at onset varied from 21 to 73 years (average 42.9 years) and included uni- and bilateral female and male BC, OVC, six men affected with prostate cancer, and a single case with pancreatic cancer. The founder haplotype did not differ between the self-identified patients representing the Afrikaner and Coloured groups.

Female *BRCA1/2* mutation carriers are at significantly increased risk for BC, OVC, and pancreatic cancer. In contrast, male mutation carriers are at increased risk for breast, prostate, and pancreatic cancer, among other types (38, 39). The benefit of targeted genetic testing of affected patients is encompassed in identifying healthy at-risk related family members early in life. By knowing their mutation status, individuals can take advantage of the options available in terms of screening and medical therapies and benefit from risk-reducing strategies to manage their risks (27). Over the past 20 years, our experience indicates a low uptake of carrier testing, which varies considerably between ethnic groups (**Table 1**). Individuals with an Afrikaner heritage are most inclined to opt for susceptibility testing (n=322, **Table 1**). Varied perceptions of the benefits related to cancer risk management (40) have a significant impact on the responsiveness and openness to cancer prevention using cascade testing in families. Many patients or individuals may be unaware of a family history of cancer and, therefore, do not consider genetic testing.

The SA Department of Health has recently recognized that health and the country’s development are integrally linked. The department has pledged to reform this sector, which is firmly embedded in its National Development Plan for 2030 (Our Future – make it work) (41). The department has since released clinical guidelines for BC control and management in which they set standards for optimal care and management to improve survival. This standard includes, among others, referral of all patients with BC (diagnosed <40 years) and/or OVC (<60 years) for comprehensive genetic testing of at least *BRCA1*, *BRCA2* and *Tp53* by means of NGS, and decreasing the time to presentation, diagnosis and treatment. The national implementation of these guidelines will dramatically increase the demand for genetic testing and exponentially contribute to this sector’s financial burden. By implementing more cost-effective targeted genetic testing as a first-tier screen, full advantage will be taken of the budget available.

These obstacles were recently addressed by the development of a novel rapid *BRCA1/2* POC assay aimed at improving the clinical management of patients with BC and associated co-morbidities (https://gtr.ukri.org/projects?ref=103993). As a more cost-effective alternative than the current assay, the ParaDNA BRCA 1.0 Research Kit using HyBeacon probes was designed. The new assay can simultaneously detect all eight recurrent SA variants in four multiplexed reactions. This assay proved to be both time- and cost-effective, although careful consideration is required before its implementation in clinical practice. The value of this innovative approach has been recognized as a future focus area when addressing personalized medicine for SA patients in both the public and private sectors.

South Africa’s extensive population diversity originated due to its geographical location with respect to historical trade routes between the east and the west, and a multi-faceted colonization history (10–14). It contributed to a unique composition, incorporating genetic signatures from Europe, Asia and Africa into SA. This diversity creates diagnostic challenges, as certain pathogenic variants are restricted to specific ethnic groups (**Table 1**). The development of an appropriate population-directed POC assay based on the results presented will help achieve the Department of Health goals to ensure optimal and standard care to all citizens. This pathology-supported genetic testing strategy was piloted by Mampunye (15) in BC patients previously referred for gene expression profiling to reduce the risk of chemotherapy overtreatment (42, 43), as well as the risk of tamoxifen resistance (44, 45).

The survey results used in the risk-assessment analysis provided valuable information and gave direction to where the ParaDNA BRCA POC assay should ideally be placed. Timeous receipt of a patients’ genotyping results may dramatically affect surgical decision-making. Receipt of a predictive, mutation-positive *BRCA1/2* POC result within an hour or on the same day was perceived as a risk by 84% of health professionals, as they thought it might be overwhelming for at-risk family members. Most healthcare professionals’ sentiment was also reflected in relation to a diagnostic test result that has implications for reproductive health and recurrence risk to offspring (81%, **Table 4**). However, this perception could be drastically influenced depending on the setting in which the test is being offered. Individual clinicians/surgeons were consulted to obtain their opinion (data not shown) before and after the results of the pilot study performed by Mampunye (15) became available. It was clear that the reaction to a positive test result on-site will differ based on the motivation for testing, namely whether it was intended for surgical decision-making or to determine familial risk.

Historically, *BRCA1/2* pathogenic variants are suspected in families with multiple women with BC and/or OVC, early ages of cancer onset, bilateral or male breast cancer. In 2001, the NCCN recommended genetic testing for patients diagnosed with breast cancer at age ≤40 (46). In 2009, however, the upper limit for age was increased in the guidelines to age 45 years (47). This evolution in guidelines demonstrates how practices change over time as new knowledge becomes available, reflecting the importance of an integrated service and multidisciplinary research model as described by Kotze et al. (48). The risk-benefit analysis supports recent suggestions to preclude relying solely on family history and pursuing the idea of testing all women diagnosed with BC or OVC for pathogenic *BRCA1/2* variants (49). The argument here is three-fold: even though these variants are relatively rare, they engender 1) high cancer risks (predictive), 2) actionable treatment targets (therapy selection), and 3) uncover inherited predisposition that may be hidden by the family structure (differential diagnosis). Some families are very small, making it difficult to recognize a strong inheritance pattern versus environmentally-induced or lifestyle-triggered genetic risk. Furthermore, in families with a male predominance, pathogenic variants may be passed through generations of men and become evident only later in female carriers. Schoeman et al. (50) reported that even in women who meet the current guidelines for genetic testing (based on family history), as few as 17.3% have been tested at a Western Cape Academic Hospital.

While clinical implementation of useful research findings may take many years, direct to the consumer applications with limited clinical utility and support have become widely available. We propose rapid founder testing supported by genetic counselling to address the associated psychosocial concerns. The survey was ideally positioned to explore some of the barriers to translation of research findings, which needs to be addressed if genomics research is to fulfil on the promises of personalized medicine (51). Barriers to *BRCA1/2* testing and extended NGS analysis include clinicians not discussing or offering testing due to a potential lack of training or knowledge, cost and insurance coverage, as well as long turn-around time of laboratory-based tests involving sample collection and transport, which all adds to the cost. Other concerns include the use of race as a proxy for risk stratification in genetics studies. This constituted an important discussion point at the SASHG conference and pre-conference workshop during which this question was addressed. Oncology specialists who expressed interest in incorporating POC *BRCA1/2* genetic testing in their cancer care pathway confirmed that they would screen all SA patients with this assay, regardless of ethnic group or language. The proposed model, which incorporates targeted genetic testing at the POC in a genomic counseling or laboratory-based near-patient setting, may overcome these barriers regardless of which of the three indications the test may be performed under, as per the clinician’s discretion.

The genetic diversity of *BRCA2* in the SA population unveiled during this investigation could potentially aid in the etiology of BC in SA, once explored, similar to the work performed by Lilyquist et al. (52). The large size of the haplotype blocks observed justifies future investigation by including polymorphic variants situated further up and downstream of the gene, together with deep intronic variants. This approach corresponds to the standard STR profiling approach. Comparing the STR (1.7 Mb) and SNP (82 kb) haplotypes for *BRCA2* c.5771_5774del and c.7934delG, showed that not all haplotypes could be distinguished when focusing on a locus spanning a relatively small genomic distance which is limited to relative conserved sequences. This was evident from the exclusion of rare minor allele variants, which could have been family or population-specific (53). The inclusion of SNPs further away from *BRCA2* might assist refining SNP haplotyping in the SA population.

This study’s significance in future investigations can be improved once a minimum of 1000 samples have been screened comprehensively. It will result in the inclusion of SNPs at a MAF > 0.001. A larger cohort will increase the p-value for variants that deviated from the Hardy-Weinberg equilibrium and justify their future inclusion in the haplotype inference. Furthermore, dividing the cohort into sub-populations before LD analysis might increase the statistical significance of the LD between SNPs, which have a low MAF in the combined population. This might increase the sensitivity of the data set for the prediction of haplotypes with a very low MAF. The continuous addition of SNP data of patients harboring these founder variants will also increase the sensitivity and accuracy of the haplotype associations. The complex diversity of the SA population observed in this study shows the need for population-based analysis performed in parallel with NGS. This will drive more appropriate population-based first-tier genotyping assays in third world countries with limited resources for pathology.

Furthermore, it could be of diagnostic significance to perform pathological association studies for each *BRCA2* haplotype with enough variation to be classified as a different *BRCA2* isoform due to the number of missense variants. As the study identified *BRCA2* SNPs in LD, together with their associated distances from each other, it could represent valuable markers for *de novo* assembly during long-range sequencing to confirm segregation patterns of novel or rare VUS. Finally, it would be important to evaluate the incidence of these variants and their impact on management in a prospective cohort of newly diagnosed breast and/or ovarian cancer patients, while comparing the results to testing strategies using local and international guidelines for founder and panel-based testing.

## Data Availability Statement

The full survey results, together with the haplotype analysis, are available at the website of the Open Genome Project (https://www.gknowmix.org/opengenome/survey/), with restricted access to supplementary data sets and analyses generated during the current study.

## Ethics Statement

The studies involving human participants were reviewed and approved by The Ethics Committee of the Faculty of Health Sciences at the University of the Free State, together with The Health Research Ethics Committee of Stellenbosch University approved all study procedures (UFS-HSD2019/1835/2910, UFS-HSD2020/0194/3006, US-N09/08/224) and the NHLS permitted use of the data. Written informed consent to participate in this study was provided by each participant.

## Author Contributions

NCM authored the original draft of this publication and obtained ethics approval. JO, PB, and NCM collated the NGS data and performed the haplotype analysis. JO performed the statistical analyses. NM and MK assisted with the risk-benefit analysis. MK, NM, JO, and EM provided critical feedback and assisted with shaping the final version of the manuscript. NCM, JO, and MK contributed significantly to the conception of the idea on which this manuscript is based. All authors contributed to the article and approved the submitted version.

## Funding

Research reported in this publication was supported by the South African Medical Research Council (vd MerweNC2013) with funds received from the South African Department of Science and Innovation (S006652, S003665), the Cancer Association of South Africa, and the National Health Laboratory Service Research Trust (GRANT004-93882; GRANT004-94366; GRANT004-94611). We also acknowledge the South African BioDesign Initiative of the Department of Science and Technology and the Technology Innovation Agency for funding the pre-conference workshop of the South African Society of Human Genetics where the survey was conducted (grant number 401/01). The funding bodies were not involved in the study design, collection, analysis and interpretation of data and writing of the manuscript.

## Conflict of Interest

MK is a non-executive director and shareholder of Gknowmix (Pty) Ltd. that is involved with the development of the POC 1.0 BRCA Research Assay.

The remaining authors declare that the research was conducted in the absence of any commercial or financial relationships that could be construed as a potential conflict of interest.
